# Diagnostic value of renal biopsy in anti-phospholipase A2 receptor antibody-positive patients with proteinuria in China

**DOI:** 10.1038/s41598-024-53445-x

**Published:** 2024-02-05

**Authors:** Shan Lu, Jing Xiao, Dong Liu, Yan Zhang, Yijun Dong, Zhanzheng Zhao

**Affiliations:** Department of Nephrology, the First Affiliated Hospital of Zhengzhou University, No. 1 Jianshe Road, Zhengzhou, 450052 China

**Keywords:** Renal biopsy, Membranous nephropathy, Anti-PLA2R antibody, Diseases, Nephrology

## Abstract

Renal biopsy remains the gold standard for diagnosing membranous nephropathy (MN). Recent studies have suggested that renal biopsy can be replaced with the serum phospholipase A2 receptor (PLA2R) antibody test for MN diagnosis in patients with nephrotic syndrome. However, this test has not been validated in the Chinese population. In this study, we investigated whether renal biopsy provides additional diagnostic information on patients with proteinuria who are seropositive for PLA2R antibodies (SAb +). We retrospectively reviewed the clinicopathological characteristics of SAb + adult patients (aged ≥ 18 years) with proteinuria (≥ 0.5 g/24 h) assessed at the Department of Nephrology, the First Affiliated Hospital of Zhengzhou University, from June 2021 to March 2022. Among a total of 801 SAb + patients who received renal biopsy, those with incomplete pathological data, diabetes or any potential cause of secondary MN were excluded. Among the 491 remaining patients, 474 had primary MN (PMN), 16 had atypical MN (AMN, 9 patients with “full house” and 2 patients with HBsAg + /HBcAg + immunofluorescence results), and 1 had focal segmental glomerulosclerosis. In patients with an eGFR of ≥ 60 mL/min/1.73 m^2^ (n = 451), 436 had PMN, and 71 (16.3%) exhibited additional biopsy findings, with obesity-related glomerulopathy being the most common. In patients with an impaired eGFR (n = 40), 38 had PMN, and 31 (81.6%) showed additional findings, with acute tubular injury being the most common. In conclusion, anti-PLA2R antibody positivity is highly predictive of PMN in Chinese adults but often coexists with other pathological diagnoses. The advantages of renal biopsy for detecting other pathologies should be weighed against the potential risks of the biopsy procedure.

## Introduction

Membranous nephropathy (MN) is the primary cause of nephrotic syndrome^1,2^. Its prevalence in the Chinese population has increased annually, making MN the most common pathological diagnosis in adults with primary glomerulonephritis^3^. This increase in MN prevalence may be due to several factors, including environmental pollution, an aging population and increased MN incidence in younger individuals^4^. At our center^5^, MN has been the most common pathological diagnosis, accounting for 24.96% of 34,630 patients undergoing a renal biopsy, followed by IgAN (24.09%).

Approximately 70–80% of primary membranous nephropathy (PMN) cases are caused by circulating autoantibodies against the M-type phospholipase A2 receptor (PLA2R) on the surface of podocytes^6^. A previous study showed that serum anti-PLA2R antibodies had a specificity of up to 99% and a sensitivity of 70–80%^6–8^. At our center^9^, the specificity and sensitivity of this test were 97.3% and 65.3%, respectively. Renal biopsy remains the gold standard for the diagnosis of MN. In addition to increasing MN detection rates, biopsy allows tissue damage evaluation and facilitates the diagnosis of other pathologies. However, renal biopsy is expensive and cumbersome and can cause several complications. Thus, in some patients^2,10^, renal biopsy may be avoided in patients who are positive for serum PLA2R antibody (SAb +).

In 2019, Bobart et al.^10^ found that among patients with MN with positive serum PLA2R antibodies, normal renal function, and negative workup for secondary causes of MN, a renal biopsy only confirmed the MN diagnosis but did not provide additional information that directly influenced clinical management. Later, the same group expanded the study to include more research centers and clinical cases while excluding patients with diabetes^2^. Consistent with their previous findings, they concluded that a renal biopsy was not needed to diagnose PMN for such patients. In another study by Wiech et al.^11^, a renal biopsy did not alter the diagnosis of PMN in SAb + patients with a normal eGFR. Moreover, the Kidney Disease: Improving Global Outcomes (KDIGO) working group suggested in 2021^12^ that renal biopsy is not needed for the diagnosis of MN in patients with nephrotic syndrome who were SAb + . However, in these studies^2,10,11^, the participants were of Western European descent, with only a few Asians included.

Therefore, we aimed to analyze the clinicopathological characteristics of adult Chinese patients who were SAb + and underwent renal biopsy and investigate the diagnostic value of renal biopsy in this population.

## Materials and methods

### Sample and data collection

Patients who underwent serum anti-PLA2R antibody tests at the First Affiliated Hospital of Zhengzhou University from June 2021 to March 2022 were retrospectively reviewed. These tests were performed due to the presence of proteinuria (≥ 0.5 g/24 h). Adult patients (aged ≥ 18 years) who were SAb + and underwent renal biopsy were selected for the study. The Medical Ethics Committee of the First Affiliated Hospital of Zhengzhou University approved the protocol for this retrospective study and waived the requirement for informed consent (protocol code 2022-KY-1152-002, date of approval: 19 October 2022). All methods in our study were conducted in accordance with relevant guidelines and regulations.

Patients’ demographic characteristics, kidney function, proteinuria, hemoglobin level, and serum albumin level at presentation were recorded. Kidney function was assessed by measuring the serum creatinine level and determining the eGFR using the Chronic Kidney Disease Epidemiology Collaboration (CKD-EPI) formula. Medical records were reviewed for results indicating diabetes and suggestive of secondary MN (e.g., malignancy, autoimmune disease, infectious disease, or monoclonal gammopathy). Patients were grouped and analyzed according to kidney function: eGFR ≥ 60 or < 60 mL/min/1.73 m^2^.

### Enzyme-linked immunosorbent assay (ELISA)

Serum anti-PLA2R antibody was measured using ELISA (EUROIMMUN Medizinische Labordiagnostika AG, D-23560 Lubeck, Seekamp 31, Germany). The test results were considered negative or positive when the serum anti-PLA2R antibody level was < 14 or ≥ 14 RU/mL^7^.

### Kidney biopsy

Kidney biopsy samples were prepared and evaluated according to standard light microscopy techniques (staining with HE, PAS, Masson, PASM + Masson), immunofluorescence microscopy, immunohistochemical staining, Congo red staining and electron microscopy (EM). The diagnosis of PMN was made following the KDIGO guidelines for glomerulonephritis. The immunofluorescence tests included IgG, IgM, IgA, C3, C4, C1q, κ, λ and IgG subtypes (IgG1-4). PLA2R was examined by indirect immunofluorescence (rabbit antibody, Sigma, USA). The immunohistochemical staining included HBsAg, HBcAg, Amyloid A and Amyloid P.

MN was classified into stages I to IV by characterizing deposits on EM according to the Ehrenreich-Churg staging system^13^. Additional pathological findings included the presence of crescents, IgA nephropathy (IgAN), obesity-related glomerulopathy (ORG), acute tubular injury (ATI), ischemic renal injury (IRI), acute/subacute tubulointerstitial nephropathy (ATIN/SATIN), and thrombotic microangiopathy (TMA). A diagnosis of atypical membranous nephropathy (AMN) was made when MN showed cellular proliferation and electron-dense deposits at multiple sites but without a definite etiology.

### Statistical analyses

Data are presented as the mean ± standard deviation for continuous data and as percentages for categorical variables. Independent t tests or Mann–Whitney U tests were used for continuous variables with normal and nonnormal distributions, respectively. Categorical data were analyzed using the chi-square test. Statistical significance was defined as P ≤ 0.05. All statistical analyses were performed using SPSS Statistics 25 (IBM Corp., Armonk, NY, USA).

### Ethics declarations

The Medical Ethics Committee of the First Affiliated Hospital of Zhengzhou University approved this retrospective study and waived the requirement for informed consent (protocol code 2022-KY-1152-002, date of approval: 19th Oct. 2022). All methods in our study were carried out in accordance with relevant guidelines and regulations.

## Results

**Figure 1:**
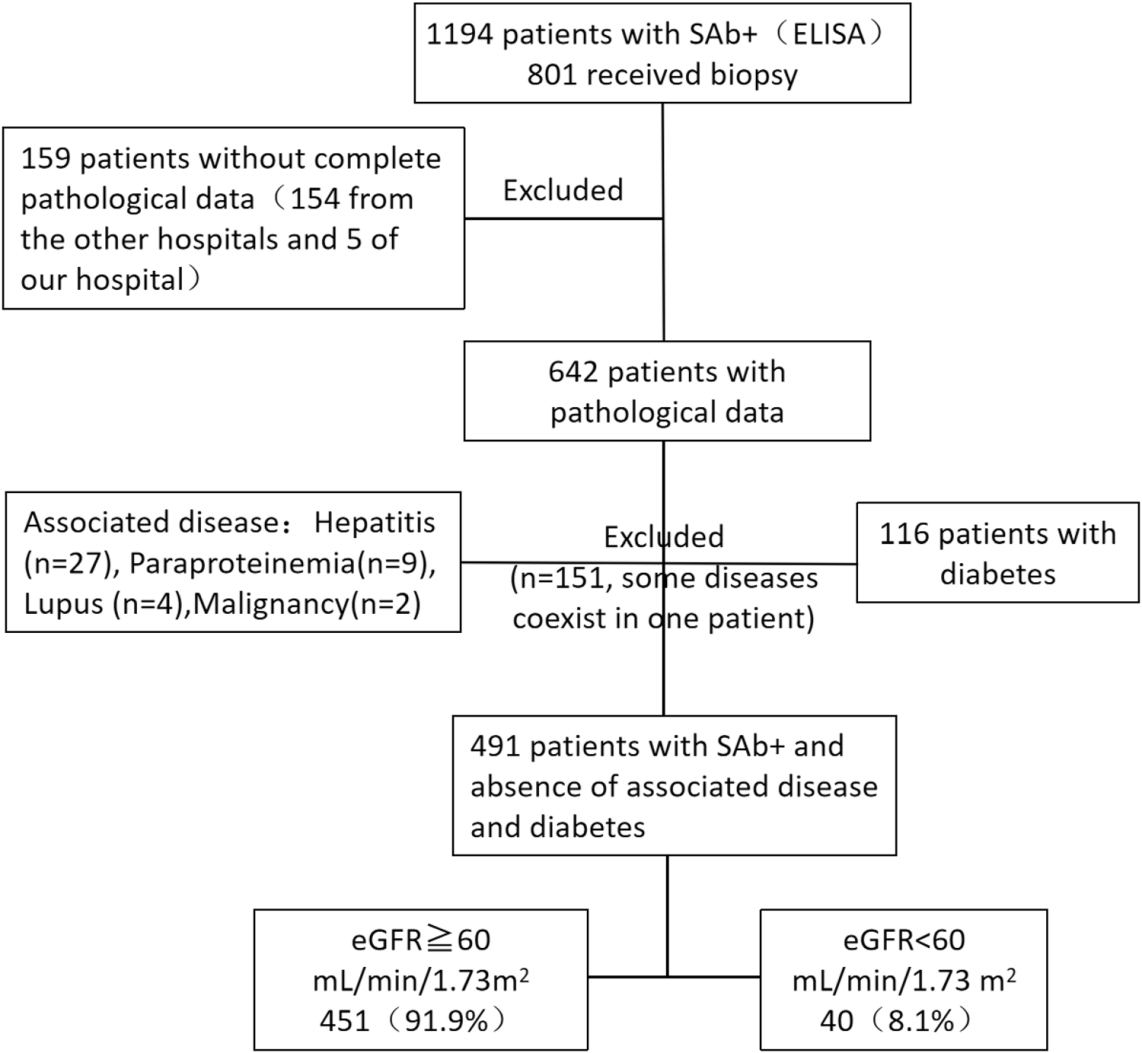
Patients with a positive PLA2R antibody test.

A total of 1194 adult patients showed SAb positivity; of these, 801 patients underwent renal biopsy. Biopsies were performed on 647 and 154 patients in our hospital and other hospitals, respectively. After excluding patients without complete pathological data, 642 patients remained (Fig. 1). Furthermore, 1 patient (0.2%) was diagnosed with FSGS, and 641 patients (99.8%) were diagnosed with MN. In addition, 116 patients had diabetes, of whom 19 were diagnosed with diabetic nephropathy.

The final cohort included 491 patients who did not have associated diseases or diabetes. When stratified by the eGFR, 451 patients (91.9%) had preserved kidney function, and 40 patients (8.1%) had impaired renal function (Fig. 1).

### Clinicopathological characteristics of SAb + patients without associated diseases or diabetes (Table 1)

**Table 1:** Clinicopathological characteristics of SAb + patients without associated diseases or diabetes.

Clinicopathological parameter	All Patients (n = 491)	eGFR≧60 mL/min/1.73 m^2^ (n = 451)	eGFR < 60 mL/min/1.73 m^2^ (n = 40)	P value
Men, n (%)	341(69.5)	311(69.0)	30(76.3)	0.427
Age, years	48.38 ± 13.15	47.92 ± 12.96	53.65 ± 14.19	0.008
SAb level (RU/mL)	143.43 ± 178.45	141.87 ± 177.17	161.00 ± 193.87	0.516
GAg positive, % (no.)	96.4(345/358)	97.0(318/328)	90.0(27/30)	0.15
IgG4 immunofluorescence assay positive, % (no.)	99.8(447/448)	99.8(414/415)	100(33/33)	1.00
Hemoglobin (g/L)	130.99 ± 19.39	132.60 ± 18.36	112.99 ± 21.75	0.000
Serum creatinine (µmol/L)	84.82 ± 77.55	71.78 ± 16.98	231.82 ± 219.33	
eGFR (mL/min/1.73 m2)	95.69 ± 24.19	110.79 ± 17.06	38.21 ± 17.56	
Serum albumin (g/L)	25.29 ± 8.07	25.47 ± 8.24	23.31 ± 5.59	0.105
Proteinuria (g/24 h)	6.05 ± 4.72	5.72 ± 4.47	9.84 ± 5.82	0.000
PMN, n (%)	474 (96.5)	436 (96.7)	38(95.0)	0.917
AMN, n (%)	16 (3.3)	15 (3.3)	1(2.5)	1.000
FSGS, n (%)	1(0.2)	0(0.0)	1 (2.5)	

*eGFR* estimated glomerular filtration rate, *SAb* serum PLA2R antibody, *GAg* glomerular deposits of PLA2R antigen, *PMN* primary membranous nephropathy, *AMN* atypical membranous nephropathy, *FSGS* focal segmental glomerulosclerosis.

Patients with impaired renal function were significantly older and had lower hemoglobin and higher protein levels than those with normal kidney function (*P* < 0.05). Among 491 patients who were SAb + without associated diseases or diabetes, 1 patient was diagnosed with FSGS, and 490 were diagnosed with MN, which included PMN (n = 474) and AMN (n = 16, 9 with “full house” and 2 with HBsAg + /HBcAg + immunofluorescence results) cases (Table 1).

### Pathological characteristics of PMN patients with SAb + and without associated diseases or diabetes (Table 2)

**Table 2:** Pathological characteristics of PMN patients with SAb + and without associated diseases or diabetes.

PMN	All Patients			P value
n (%)	(n = 474)	eGFR≧60 mL/min/1.73 m^2^(n = 436)	eGFR < 60 mL/min/1.73 m2(n = 38)	
Stage I	8(1.7)	6(1.4)	2(5.3)	0.020
Stage II	278(58.6)	270(61.9)	8(21.1)	
Stage III	175(36.9)	150(34.4)	25(65.8)	
Stage IV	13(2.7)	10(2.3)	3(7.9)	
Additional biopsy findings, n (%)	102(21.5)	71(16.3)	31(81.6)	0.000
IgAN		17(3.9)	0(0)	0.383
ORG		23(5.3)	2(5.3)	1.000
ATI		7(1.6)	11(28.9)	0.000
ATIN/SATIN		6(1.4)	6(15.8)	0.000
IRI		7(1.6)	5(13.2)	0.000
Others		IgAN with IRI(n = 1) IgA deposition(n = 6) Endothelial cell injury(n = 2) TMA(n = 2)	ATI with IRI(n = 3) SATIN with IRI(n = 1) IgA deposition(n = 1) Crescents(n = 2)	

*ORG* obesity-related glomerulopathy, *ATI* acute tubular injury, *IRI* ischemic renal injury, *ATIN/SATIN* acute tubulointerstitial nephropathy/subacute tubulointerstitial nephropathy, *TMA* thrombotic microangiopathy.

In patients with a decreased GFR, the pathological grading and proportion of additional biopsy findings were higher than those in patients with preserved kidney function (P < 0.05). The most common additional biopsy finding was ORG in patients with preserved kidney function and ATI in those with impaired kidney function. Patients with renal impairment also had a higher proportion of other findings, including ATI, IRI, and ATIN/SATIN (Table 2).

### Clinical characteristics of PMN or AMN patients and PMN patients with or without ORG/IgAN who were SAb + with no associated diseases or diabetes

Patients with PMN had lower serum albumin levels than patients with AMN (*P* < 0.05) (Table 3). PMN patients with ORG (*P* < 0.05) had higher hemoglobin levels (Supplemental Table S1). The SAb titer was lower in PMN patients with IgAN than in those without IgAN (*P* < 0.05) (Supplemental Table S2).

**Table 3:** Clinical characteristics of patients with PMN or AMN who were SAb + with no associated diseases or diabetes.

Clinical parameter	PMN (n = 474)	AMN (n = 16)	P value
Men, n (%)	327 (69.0)	13 (81.3)	0.441
Age, years	48.64 ± 12.96	42.81 ± 15.7	0.080
SAb level (RU/mL)	143.28 ± 178.81	155.62 ± 175.71	0.786
Hemoglobin (g/L)	130.97 ± 19.34	130.54 ± 21.29	0.932
Serum creatinine (µmol/L)	84.76 ± 78.46	76.93 ± 26.62	0.691
eGFR (mL/min/1.73 m2)	95.72 ± 23.97	99.20 ± 25.80	0.569
Serum albumin (g/L)	25.06 ± 6.06	32.73 ± 30.01	0.000
Proteinuria (g/24 h)	6.00 ± 4.72	7.08 ± 4.26	0.364

## Discussion

Anti-PLA2R antibody testing has improved the diagnosis and treatment of MN^6–8^. Anti-PLA2R antibodies are correlated with MN progression, remission, and recurrence. In a study by Bobart et al.^2^, among 101 SAb + patients without associated diseases or diabetes, all renal biopsies indicated the presence of MN. Only 12 patients (11.9%) showed copathologies, which did not significantly affect the diagnosis and treatment. In our study, among the 491 SAb + patients without associated diseases or diabetes, 1 patient was diagnosed with FSGS, 490 (99.8%) were diagnosed with MN, and 16 of them were diagnosed with AMN; additionally, 21.5% of patients with PMN exhibited additional biopsy findings. These results suggest that serum anti-PLA2R antibody testing is highly accurate for diagnosing MN in Chinese adults; however, additional biopsy findings were more frequent in the Chinese population than in the Western population^2,10,11^. Thus, the decision to replace renal biopsy with the serological antibody test in Chinese patients should be weighed against potential complications of the biopsy procedure.

As serum anti-PLA2R antibodies can spontaneously diminish over time, staining of glomerular deposits of PLA2R antigen (GAg) has been found to have higher sensitivity for diagnosing MN^9^. In a study by Peihong^14^, patients who were SAb − /GAg − were more likely to exhibit complete remission than those who were SAb − /GAg + or SAb + /GAg + . Recently, Luo et al.^15,16^ reported that a small subpopulation of SAb + patients with MN was negative for GAg. These patients showed higher SAb titers, worse clinicopathological manifestations and poorer treatment response and prognosis, indicating a distinct clinical subtype of PMN. The specific etiology and pathogenesis of SAb + /GAg − remain unknown, but SAb + /GAg − may occur when anti-PLA2R antibodies are not pathogenic or when epitopes are poorly accessible at the time of renal biopsy; it may also be a technical artifact^16^. In our study, 18 patients (4.0%) were negative for GAg. Although the GAg status did not affect clinical decisions regarding treatment, it may be explored further for prognosis.

In recent years, a new type of MN has been increasing in China. It is characterized by mesangial proliferation and electron-dense deposits at multiple sites. The immunofluorescence results of most of these patients demonstrate a “full house” pattern of MN, which indicates positivity for IgG, IgA, IgM, C3 and C1q, but without a definite etiology^17^. These cases have been referred to as atypical MN, lupus-like MN or “full house” MN. In our study, 9 of the 16 patients with AMN (3.3%) showed a “full house” pattern. Previous work^18^ suggested that this might be SLE confined to the kidney. However, follow-up studies^19,20^ demonstrated that extrarenal manifestations of SLE in these patients only occurred rarely or not at all. Sam et al.^21^ hypothesized that lupus-like MN might be a new type of MN whose baseline characteristics and prognosis fall between those of PMN and LN-MN. However, Jiang Z et al.^17,19^ conducted a study in the Chinese population and suggested that AMN is merely a pathological variant of MN because there was no difference in SAb titers, clinical manifestations and renal outcomes between patients with PMN and AMN. Thus, current evidence suggests that the diagnosis of AMN may not affect treatment decisions, but a renal biopsy could be helpful in distinguishing different types of MN. Further studies on AMN and its clinical consequences are warranted.

Two AMN patients were positive for HBsAg/HBcAg staining but were serologically negative for HBV-MN. The current prevalence of HBV infection in the general Chinese population is 5–6%, with approximately 70 million patients with chronic HBV infection^22^. Approximately 3–5% of patients with HBV develop kidney disease, with HBV-MN as the most common histological change^23^. Most patients with HBV-associated glomerulonephritis (HBV-GN) have a history of chronic HBV infection and are seropositive for HBsAg. However, our previous work and several other studies^24–26^ demonstrated that some HBV-GN patients (12–37.6%) are seronegative for HBV markers. This could be due to the delay in the recovery of glomerulonephritis relative to the resolution of HBV infection or to occult hepatitis B virus infection. We found similar treatment remission rates for HBV-MN and PMN in our previous study^24^. Moreover, HBV-GN remission was similar in both serum HBsAg + and HBsAg − patients^25^. Thus, HBsAg/HBcAg staining may be a crucial test in Chinese patients with MN.

In our study, 18 PMN patients (3.7%) had superimposed IgAN, which is also the most common renal pathological finding in Asians^27^. At our center^5^, the proportion of MN with IgAN was 1.3% in total renal pathology, which was higher than that reported in Western studies^10,11^. In some studies^28–31^, a small proportion of Chinese patients with MN also reported exhibiting IgAN. To date, the etiology of MN complicated by IgAN is unknown. This entity may be due to the superposition of two independent pathologies. However, some researchers^28^ have suggested that either PMN or IgAN can be a primary event that induces the occurrence of another kidney disease. In these studies^28,29^, patients with MN + IgAN displayed similar clinical features as MN patients but milder pathological lesions than IgAN patients. Furthermore, MN + IgAN patients showed similar treatment responses but higher cumulative incidence rates of remission than MN patients. In our study, mild mesangial proliferation with IgAN was more common, which is consistent with previous studies. We also found that the SAb titer was lower in PMN + IgAN patients, suggesting that there could be more MN variants in patients with mild seropositivity.

In our study, ORG (5.1%) was found to be the most common additional disease. Studies on the relationship between obesity and MN are scarce. Only one study^32^ demonstrated that obesity may play an essential role in mesangial lesions of PMN. Obesity has currently become an epidemic. ORG is strongly associated with CKD^33^, and patients with obesity are likely to develop drug-induced diabetes when treated with steroids or immunosuppressants, which are the mainstay for MN therapy. Therefore, a renal biopsy could be highly considered for SAb + patients with obesity.

In our study, patients with impaired GFR exhibited additional findings (76.3%) more frequently than those with preserved kidney function (P < 0.05). This is consistent with data reported by Bobart et al.^2,10^^.^ ATI was the most common superimposed disease, followed by ATIN/SATIN, which could be the main cause of renal impairment in MN patients in our study. The presence of ATIN/SATIN and crescents in patients with a decreased GFR should prompt the consideration of more aggressive treatment. Moreover, the pathological grading of PMN was higher in the low-GFR group than in the normal-GFR group. However, previous studies^34,35^ demonstrated that creatinine clearance in PMN patients was related to the chronicity of renal pathology. Furthermore, the PMN stage determined via EM could not predict kidney survival. Thus, more studies are warranted to elucidate the significance of the PMN stage. For SAb + patients with renal insufficiency, renal biopsy can reveal the underlying cause of renal impairment.

We previously^9^ demonstrated that there is no difference in the specificity (97.3%) for diagnosing MN via ELISA using different cutoff values of 14, 20 and 40 RU/mL. However, the sensitivity was the highest when the cutoff value was 14 RU/mL. Porcelli et al.^36^ also suggested that 14 RU/mL is the optimal cutoff value. In contrast, Bobart et al. used a cutoff value of 20 RU/mL^2,10^. In our study, an 18-year-old patient with an SAb titer of 17 RU/mL had FSGS with clinical manifestations of nephrotic syndrome complicated by AKI. Similar results^8,37^ have been reported in other studies; SAb + patients with low SAb titers exhibited biopsy-proven FSGS. Although the relationship between FSGS and MN remains unclear, the lower titer of SAb in this patient did not seem to be pathogenic. A higher cutoff value of the SAb titer could help achieve an accurate diagnosis, and ELISA and indirect immunofluorescence techniques are comparable and complementary. It is best to perform a renal biopsy in a patient with a low SAb titer (< 20 RU/mL), especially in younger patients.

This study has some limitations. Although more patients were included in the current study than in the previous study, data were collected retrospectively from a single center and were limited to those available in the electronic medical records. Second, the lack of follow-up data limits the determination of the prognostic value of this test. This question is currently being addressed in our follow-up studies.

In summary, our study demonstrated that anti-PLA2R antibody testing has high accuracy for noninvasively diagnosing PMN in a large Chinese cohort. Compared with studies in Western populations^2,10,11^, we observed pathological variations in MN and complicating diseases more frequently in the Chinese population. Thus, an individualized approach should be employed when deciding whether to use antibody testing instead of renal biopsy to diagnose MN in Chinese patients. We recommend that in patients with nephrotic syndrome, preserved kidney function, positive serum anti-PLA2R antibody (≥ 20 RU/mL; ELISA), and no comorbidities, renal biopsy is not necessary for the diagnosis of MN. However, close observation and follow-up are warranted to monitor the emergence of new clinical manifestations, immunological seropositivity and poor treatment response in these patients. Furthermore, renal biopsy should be performed to avoid misdiagnosis or missed diagnoses.

### Supplementary Information

Supplementary Tables.

## Data Availability

Datasets are available from the corresponding author upon reasonable request.
