# Supplementary material for: Diagnostic value of renal biopsy in anti-phospholipase A2 receptor antibody-positive patients with proteinuria in China
Source: Sci Rep. 2024 Feb 5;14:2907. doi: 10.1038/s41598-024-53445-x (PMC10844597; doi:10.1038/s41598-024-53445-x)
Supplement: Supplementary file 1 — Supplementary Tables. [file 41598_2024_53445_MOESM1_ESM.docx]

**Supplemental Table 1**. **Clinical characteristics of PMN patients with or without ORG who had SAb+ and absence of associated diseases or diabetes.**

| Clinical parameters | PMN without ORG (n=465) | PMN with ORG (n=25) | P-value |
| --- | --- | --- | --- |
| Men,n (%) | 320(68.8) | 20(80.0) | 0.237 |
| Age, years | 48.63±13.14 | 44.96±11.69 | 0.172 |
| SAb level (RU/mL) | 141.24±172.71 | 189.18±175.71 | 0.191 |
| Hemoglobin (g/L) | 130.31±19.34 | 142.85±16.29 | 0.002 |
| Serum creatinine (umol/L) | 84.09±77.64 | 92.12±72.02 | 0.614 |
| eGFR (mL/min/1.73 m2) | 95.89±23.97 | 94.80±25.14 | 0.826 |
| Serum albumin (g/L) | 25.34±8.21 | 24.81±4.48 | 0.751 |
| Proteinuria (g/24 h) | 5.99±4.62 | 6.90±6.07 | 0.346 |

**Supplemental Table 2. Clinical characteristics of PMN patients with or without IgAN who had SAb+ and absence of associated diseases or diabetes.**

| Clinical parameters | PMN without IgAN (n=472) | PMN with IgAN (n=18) | P-value |
| --- | --- | --- | --- |
| Men,n(%) | 327(69.3) | 13(72.2) | 0.790 |
| Age, years | 48.42±13.22 | 49.06±9.19 | 0.781 |
| SAb level (RU/mL) | 146.60±180.98 | 67.23±55.41 | 0.000 |
| Hemoglobin (g/L) | 130.97±19.60 | 130.42±12.79 | 0.906 |
| Serum creatinine (umol/L) | 84.91±78.69 | 73.87±15.73 | 0.553 |
| eGFR (mL/min/1.73 m2) | 95.71±24.29 | 99.19±14.99 | 0.547 |
| Serum albumin (g/L) | 25.37±8.16 | 23.72±4.84 | 0.393 |
| Proteinuria (g/24 h) | 6.10±4.76 | 4.11±2.63 | 0.078 |
